# Extracellular matrix: Brick and mortar in the skeletal muscle stem cell niche

**DOI:** 10.3389/fcell.2022.1056523

**Published:** 2022-11-29

**Authors:** Svenja C. Schüler, Yuguo Liu, Simon Dumontier, Michel Grandbois, Emmeran Le Moal, DDW Cornelison, C. Florian Bentzinger

**Affiliations:** ^1^ Département de Pharmacologie-Physiologie, Faculté de Médecine et des Sciences de la Santé, Institut de Pharmacologie de Sherbrooke, Centre de Recherche du Centre Hospitalier Universitaire de Sherbrooke, Université de Sherbrooke, Sherbrooke, QC, Canada; ^2^ Division of Biological Sciences Christopher S. Bond Life Sciences Center, University of Missouri, Columbia, MO, United States

**Keywords:** muscle stem cells, satellite cells, extracellular matrix, stem cell niche, skeletal muscle, MuSCs

## Abstract

The extracellular matrix (ECM) is an interconnected macromolecular scaffold occupying the space between cells. Amongst other functions, the ECM provides structural support to tissues and serves as a microenvironmental niche that conveys regulatory signals to cells. Cell-matrix adhesions, which link the ECM to the cytoskeleton, are dynamic multi-protein complexes containing surface receptors and intracellular effectors that control various downstream pathways. In skeletal muscle, the most abundant tissue of the body, each individual muscle fiber and its associated muscle stem cells (MuSCs) are surrounded by a layer of ECM referred to as the basal lamina. The core scaffold of the basal lamina consists of self-assembling polymeric laminins and a network of collagens that tether proteoglycans, which provide lateral crosslinking, establish collateral associations with cell surface receptors, and serve as a sink and reservoir for growth factors. Skeletal muscle also contains the fibrillar collagenous interstitial ECM that plays an important role in determining tissue elasticity, connects the basal laminae to each other, and contains matrix secreting mesenchymal fibroblast-like cell types and blood vessels. During skeletal muscle regeneration fibroblast-like cell populations expand and contribute to the transitional fibronectin-rich regenerative matrix that instructs angiogenesis and MuSC function. Here, we provide a comprehensive overview of the role of the skeletal muscle ECM in health and disease and outline its role in orchestrating tissue regeneration and MuSC function.

## Introduction

Multicellularity is thought to have emerged independently over 25 times during the history of life on Earth (Grosberg and Strathmann, 2007). When compared to single-celled organisms, multicellularity has the advantage that cells can specialize and come together to form tissues and organs that have distinct functions. Animals (metazoans) that have developed an astonishing variety of body plans are amongst the most complex organisms in the phylogenetic tree of life. The development of an extracellular matrix (ECM), the non-cellular element of all tissues, was essential in enabling the rapid evolution of the metazoan phyla (Hynes, 2012). ECM consists of a sophisticated and highly organized network of macromolecules occupying the space between cells. The wide range of biological functions of the ECM includes providing structure to cells and tissues, serving as an adhesion substrate, facilitating the communication of neighboring cells, transmitting mechanical signals, regulating cellular growth, and promoting or restricting cell movement. The critical importance of ECM for metazoans is illustrated by the fact that heterotrimeric laminins are already synthesized by 16-cell embryos, while collagen IV is detectable in the inner cell mass of the blastocyst (Leivo et al., 1980; Cooper and MacQueen, 1983). In its basic structure, the majority of ECM is composed of two main classes of biomolecules: glycosaminoglycan (GAGs) polysaccharide chains, which are typically linked to protein in the form of proteoglycans, and proteinaceous ECM components such as collagen, fibronectin, and laminin. Most ECM proteins are composed of multiple, often repeated domains, some of which are highly evolutionarily conserved.

In metazoans, the ECM of the basal lamina is defined by its specialized, flat laminar structure and anatomic location at the interface of parenchymal cells with the extracellular space. The basal lamina, which depending on the tissue is between 40 and 120 nm thick, includes a core network of self-assembling laminins that can form continuous sheets intertwined with a network of collagen and interconnected by linker molecules such as the proteoglycans nidogen and perlecan (Aumailley et al., 1993; Hynes, 2012). The interstitial matrix occupies the extracellular space adjacent to the pericellular basal lamina. The majority of interstitial ECM is made up of proteoglycans, which due to negatively charged sulfates in their glycosaminoglycan chains are typically highly hydrated (Frantz et al., 2010). In contrast to the network collagens found in the basal lamina, the interstitial matrix contains fibrillar collagen, which binds to proteoglycans to form a sturdy gel. Depending on the tissue, the interstitial ECM also contains fibronectin, a high-molecular weight glycoprotein with a wide spectrum of functions ranging from structural organization to cell surface receptor interactions (Singh et al., 2010).

Next to its role during development, the ECM has key functions for tissue regeneration in adult organisms. This includes both regular cellular renewal during tissue homeostasis and reparative regeneration after injury. Several metazoan phyla have a remarkable regenerative capacity that in some cases persists throughout life. For instance, urodele amphibians can regenerate entire limbs through an epimorphic regeneration process mediated by a blastema formed either from dedifferentiation or tissue-resident stem cells involving a unique pro-regenerative ECM (Sandoval-Guzman et al., 2014; Seifert and Muneoka, 2018). In adult mammals, the regenerative capacity of tissues ranges from absent to very low in the central nervous system to highly efficient in the liver, the latter of which can regrow to its original size after losing up to 70% of its original volume within 1 week in rodents (Zhao et al., 2016). By providing interaction sites and through its physical properties, the ECM presents instructive signals to cells during adult tissue regeneration, while its ability to sequester growth factors and generate concentration gradients also has indirect effects on this process (Hynes, 2009).

Many tissues in vertebrates contain adult stem cells that are involved in physiological regeneration, maintaining homeostasis as well as in reparative regeneration following injury or loss of body parts. Arguably, the most prominent example is hematopoietic stem cells that can self-maintain while producing a wide spectrum of circulating cells (Clevers and Watt, 2018). Other examples include stem cells in the skin, certain brain structures, the intestine, and skeletal muscle. While all cell types in vertebrates are exposed to some form of ECM, adult stem cell niches often contain a highly specialized instructive structural microenvironment (Gattazzo et al., 2014). ECM niches involve both autoregulatory matrix that is secreted by stem cells into their own microenvironment and molecules produced by supportive accessory cell types. Here, we discuss the ECM in the stem cell niche in the context of skeletal muscle which, since the first description of a putative myogenic progenitor cell residing on the surface of muscle fibers by electron microscopy in 1961, has become one of the most studied paradigms of adult tissue regeneration (Katz, 1961; Mauro, 1961; Scharner and Zammit, 2011).

## Skeletal muscle stem cells

Without considering “connective tissue”, an umbrella term used for ECM spaces and their associated fibroblast-like cells, skeletal muscle represents the most abundant tissue in vertebrates. Adult skeletal muscle contains a population of stem cells that are directly associated with muscle fibers referred to as “satellite cells” or “muscle stem cells” (MuSCs). Under homeostatic conditions adult MuSCs have exited the cell cycle and reside in a quiescent state under the ECM of the basal lamina that surrounds each individual muscle fiber (Mashinchian et al., 2018; Ancel et al., 2021). Following damage to muscle fibers, MuSCs can activate, enter the cell cycle and divide. While a fraction of MuSCs self-renews and maintains its stem cell characteristics, the bulk of cells progresses through the myogenic program and ultimately turns into myocytes that align and fuse to form new muscle fibers replacing the lost cells.

In everyday life, skeletal muscle tissue can be injured by blunt or penetrating traumatic events, for instance following road traffic, workplace, and sports accidents or because of lacerations during surgical interventions. Damage to muscle fibers leads to hypercontraction, necrosis, and subsequent removal of cellular debris by immune cells. In most types of skeletal muscle injury, the majority of ECM initially remains in the wound and MuSCs can migrate along the remaining scaffold to fuse and form new muscle fibers for tissue repair (Garg et al., 2015; Webster et al., 2016). Owing to MuSCs, skeletal muscle has the striking ability to go through multiple rounds of injury and repair without significant loss of functionality. However, efficient adult skeletal muscle regeneration in mammals depends on instructive signals from the existing extracellular matrix (Singhal and Martin, 2011; Webster et al., 2016). If large portions of a skeletal muscle including the ECM are lost, the tissue fails to regenerate efficiently. A well-known example of this kind of injury is volumetric muscle loss (VML) occurring after excision of large portions of skeletal muscle, for instance as a consequence of explosions on the battlefield (Testa et al., 2021). In case of VML the missing parts of damaged muscles are partially replaced by fibrotic scar tissue and revascularization and reinnervation of the small number of disoriented fibers that are forming at the injury site is inefficient. Thus, although extensive remodeling of the ECM takes place during skeletal muscle regeneration, at least in the early stages an existing ECM template is required for efficient MuSC function.

Snake venoms such as cardiotoxin or notexin, a frequently used experimental paradigm for skeletal muscle regeneration, lead to a type of myotoxic injury that induces hypercontraction and degeneration of muscle fibers but is thought to leave ECM and mono-nuclear cell types including MuSCs largely intact (Hardy et al., 2016). Thus, this type of injury induces highly coordinated and efficient skeletal muscle regeneration with MuSCs reaching a peak in proliferation around 3–5 days post injury (Fukada et al., 2022). During the first few days after snake venom injury remnants of the original basal laminae are still present in the tissue, while at later stages newly formed fibers of small diameter and their associated proliferating MuSCs are surrounded by proportionately sized basal laminae containing specialized pro-regenerative ECM molecules (Bentzinger et al., 2013). The latter observation suggests that *de novo* ECM synthesis and remodeling of the basal lamina, as well as removal of the residual scaffold during the regenerative time-course are highly coordinated processes critical to efficient skeletal muscle regeneration (Yoshimoto et al., 2020). While the cellular sources and mechanisms driving remodeling of the pro-regenerative ECM after myotoxic injury remain only partially understood, evidence points towards immune cells and fibroblast-like cell populations as major contributors to these dynamic processes. MuSCs integrate paracrine and autocrine ECM signals through several different transmembrane proteins that, together with the coordinated activation of cell-cell and growth factor receptors, instruct all stages of adult myogenesis.

## Transmembrane extracellular matrix components and receptors in muscle stem cells

**FIGURE 1:**
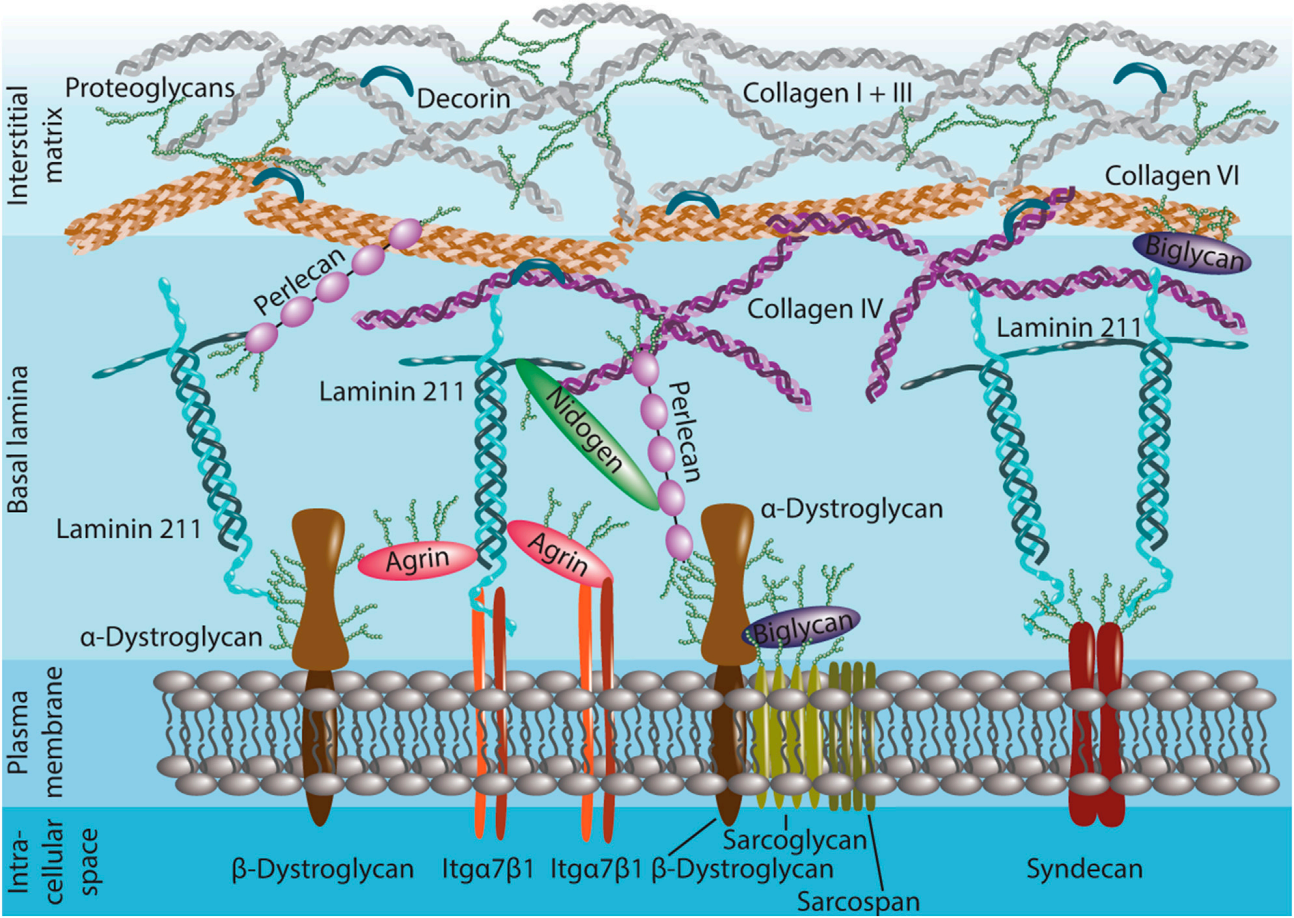
Structure of the extracellular matrix (ECM) in the quiescent muscle stem cell (MuSC) niche. MuSCs interface directly with the laminin 211 and collagen IV-rich basal lamina. Laminin 211, agrin, biglycan and perlecan interact with cell surface receptors such as dystroglycan and integrin (Itg) α7β1 and connect to syndecans. Nidogen and perlecan link the collagen IV network in the basal lamina to the plasma membrane and laminin. Perlecan, biglycan and decorin help anchoring the basal lamina to collagen I, III, and VI in the proteoglycan-rich interstitial matrix. Many ECM proteins are extensively glycosylated (green sidechains).

The basal lamina of skeletal muscle, which surrounds the muscle fiber plasma membrane and sublaminar MuSCs, is composed of a scaffold of heterotrimeric laminins and an interlaced network of collagen IV (Figure 1). Laminins that bind to integrins and dystroglycan are the predominant macromolecular polymeric ECM component expected to interface directly with MuSCs. Several other ECM components in the basal lamina can bind to cell surface receptors. This includes the non-neuronal isoform of agrin, which links the laminin network to both integrins and dystroglycan (Bezakova and Ruegg, 2003). The heparan sulfate proteoglycan perlecan, a large multidomain protein, connects the collagen IV and laminin networks to dystroglycan (Talts et al., 1999). Biglycan, a small leucine-rich proteoglycan has a role in tethering dystroglycan and some of its associated sarcoglycans to collagens (Bowe et al., 2000; Rafii et al., 2006). The basal lamina is laterally stabilized by proteins such as the nidogens that have multiple interaction partners including laminin, collagen IV, and perlecan (Zhou et al., 2022). Although nidogens can also bind to integrins and promote cell spreading, evidence for direct cell surface receptor interactions in skeletal muscle remains limited (Dong et al., 1995; Wu et al., 1995). Under homeostatic conditions, the basal lamina spatially separates MuSCs from interstitial ECM components. However, the example of the interstitial collagens I and VI that are linked to each other and the C-terminus of collagen IV in the basal lamina through the fibril growth promoting proteoglycan decorin, illustrates that all ECM layers in skeletal muscle are intimately interconnected and spatially distant structures may have indirect effects on MuSC function (Danielson et al., 1997; Kuo et al., 1997; Nareyeck et al., 2004).

**FIGURE 2:**
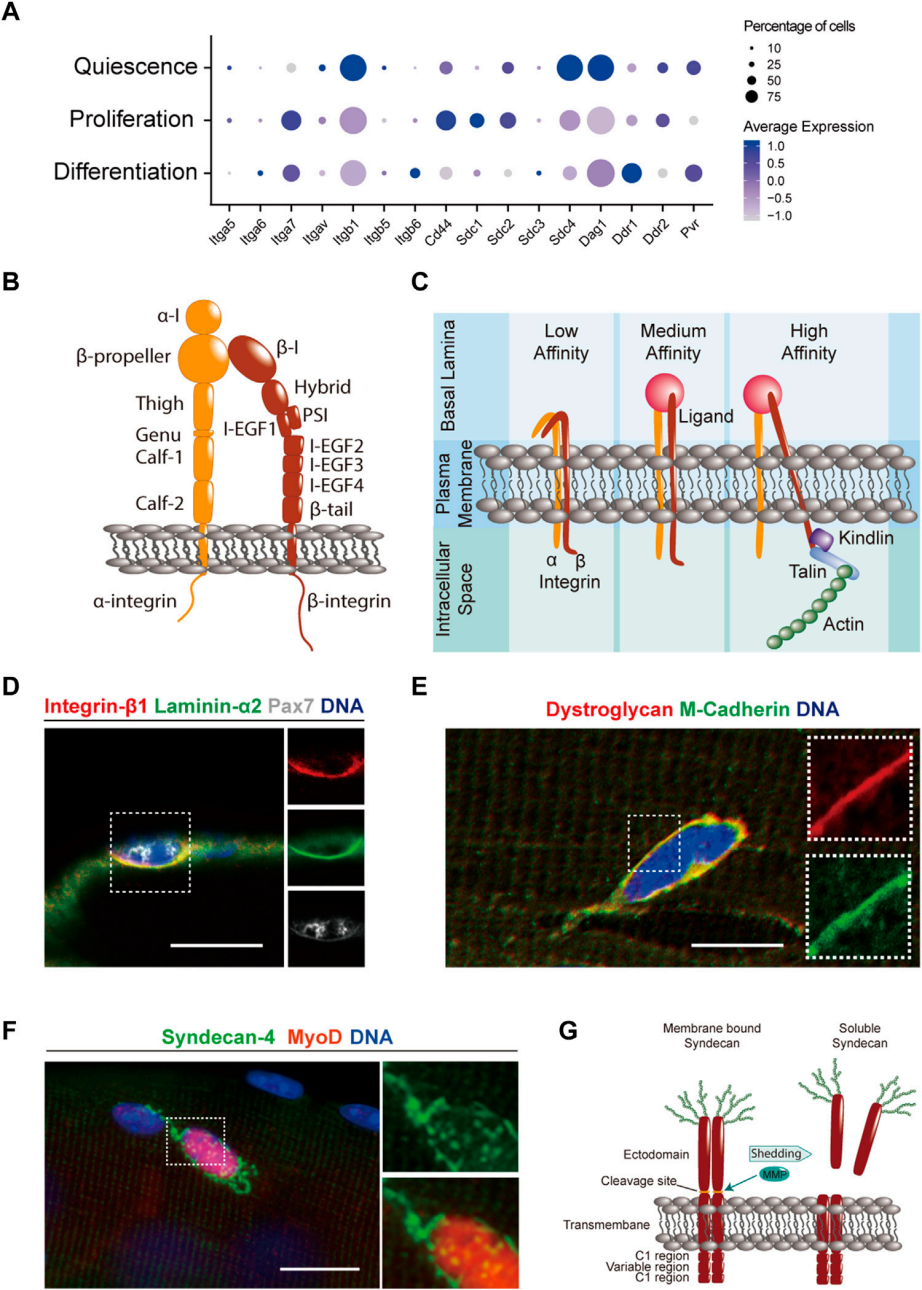
Transmembrane ECM components and receptors in MuSCs. **(A)** Dot plot of single cell RNA-sequencing data (Oprescu et al., 2020) obtained from the Gene Expression Omnibus (GEO) database (Barrett et al., 2013) from uninjured and regenerating skeletal mouse muscle at 5 days post injury that was generated using Seurat 4.0.5. (Hao et al., 2021). Genes coding for ECM interacting transmembrane proteins were selected based on the GO terms “adhesion receptor function” in the adhesome data base and “ECM receptors” in the reactome data base (Zaidel-Bar et al., 2007; Winograd-Katz et al., 2014; Griss et al., 2020). The data for quiescence was obtained from uninjured muscles, while proliferation and differentiation were defined based on the presence or absence of expression of genes such as *Pax7*, *MyoD*, *MyoG*, and *MKI67* at 5 days post injury. Only genes significantly expressed by at least 10% of MuSCs are shown. **(B)** Scheme showing the domain structure of the integrin-α and -β subunits. **(C)** Scheme illustrating how integrins transition from their low-to high-affinity state upon multivalent ligand binding and intracellular stabilization by focal adhesion molecules. **(D)** Immunostaining showing integrin β1 (red), laminin α2 (green), Pax7 (white), and DNA (blue) in a MuSC on a manually teased mouse muscle fiber bundle preparation. **(E)** Immunostaining showing dystroglycan (red), M-cadherin (green), and DNA (blue) in a MuSC on an enzymatically isolated single mouse muscle fiber. **(F)** Immunostaining showing syndecan-4 (green), MyoD (red) and DNA (blue) in MuSCs on an enzymatically isolated single mouse muscle fiber after 24 h of culture. **(G)** Scheme showing the domain structure of syndecan dimers in their membrane bound and soluble forms. Matrix metalloproteinases (MMPs) cleave membrane syndecans in a process called “shedding” and release the extracellular domain into the surrounding ECM. Scale bars = 20 μm **(D,E)**, and 10 μm **(F)**.

MuSCs contain high levels of the heterodimeric laminin receptor integrin α7β1, which has frequently been used as a marker for flow cytometry isolation (Figures 2A,D) (Blanco-Bose et al., 2001). While the mRNA coding for integrin β1 is readily detectable in quiescent MuSCs, the α7 subunit appears to be expressed at lower levels (Figure 2A). This phenomenon may be due to negative feedback regulation induced by the high abundance of laminin ligand in the quiescent niche, a notion that is supported by the fact that integrin α7 mRNA is upregulated in activated or differentiating cells when the ECM surrounding the cells becomes more heterogenous. The integrin *α* subunit consists of a short cytoplasmatic tail, followed by a transmembrane domain, two β-sandwich “calf” domains, the genu or “knee” domain, and an Ig-like “thigh” domain supporting the β-propeller that forms the ligand binding head (Figure 2B) (Gahmberg et al., 2009). The integrin β subunit is composed of a cytoplasmatic tail, followed by a transmembrane segment, the β-tail, 4 EGF-like domains, a β I-like domain, and the plexin-semaphorin-integrin (PSI) domain. Both the *α* and β integrin subunits contain binding sites for the divalent metal ions Mg^2+^ and Ca^2+^ that have differential functions in regulating ligand affinity (Zhang and Chen, 2012). However, complete removal of these cations using EDTA leads to an inhibition of integrin-ligand interactions, a mechanism that is commonly exploited when mild enzyme-free dissociation buffers are used to detach primary MuSC derived myoblasts from culture dishes containing ECM coating. Integrins have different ligand affinity states that depend on their structural conformation (Figure 2C) (Takagi and Springer, 2002). In their inactive state the two integrin subunits remain in a bent over, low-affinity conformation. Following intracellular signals or binding to ECM the extracellular portion of the two subunits can extend but remains in a closed low-ligand-affinity conformation. Upon multivalent ligand binding and intracellular stabilization by focal adhesion molecules including kindlin and talin that are anchored to the actin cytoskeleton, integrins will transition to the open high-affinity state in which the cytoplasmic “leg” domains become separated. Pax7-dependent deletion of integrin β1 leads to a depletion of the MuSC pool, suggesting a role in maintaining quiescence (Rozo et al., 2016). Interestingly, aged MuSCs contain less active, high-affinity integrin β1, and treatment with an activating antibody ameliorates age-associated stem cell dysfunction. Integrin α7 can be alternatively spliced to generate the variants α7X1, α7X2 that bind to different forms of laminin (von der Mark et al., 2002). α7X1, which does not contain exon 6 of the *ITGA7* gene, appears to be mainly expressed during skeletal muscle development, while α7X2, which does not contain exon 5, is expressed in adult skeletal muscle (Collo et al., 1993; von der Mark et al., 2002). Inclusion of exon 5 leading to α7X1 expression at the expense of α7X2 impairs asymmetric MuSC division and the generation of committed myogenic progenitors (Dominici and Richard, 2022). Following activation, MuSCs have been reported to upregulate integrin α6β1, which binds to certain cell-autonomously secreted laminin isoforms driving planar divisions required for expansion of the stem cell pool (Rayagiri et al., 2018). While single cell sequencing data suggests that MuSCs *in vivo* do not express high levels of collagen-binding integrin heterodimers, it has been shown that primary MuSC-derived myoblasts cultured on collagen-rich substrates express a variety of *α* and *β* subunits including the collagen receptors integrin α1β1 and α2β1 (Figure 2A) (Hynes, 2002; Siegel et al., 2009). Interestingly, integrin α7β1 also has a role in MuSC motility, and antibody mediated blockade of either subunit slowed the movement of cells on cultured single fibers in live-imaging experiments. In contrast, blockade of the RGD specific integrin α5 and laminin specific integrin α6 subunits increased MuSC motility suggesting an inhibitory effect of certain ECM components on cell migration (Siegel et al., 2009).

The second highly expressed ECM receptor in MuSCs is dystroglycan (Figures 2A,E). Dystroglycan is synthesized from a single gene (*DAG1*) and is post-translationally cleaved into *α* and *β* subunits (Figure 1) (Ibraghimov-Beskrovnaya et al., 1992). *α*-dystroglycan is peripherally linked to β-dystroglycan, which spans the plasma membrane and is directly connected to the inner membrane protein dystrophin (Winder, 2001). Dystroglycan is a core component of the dystrophin glycoprotein complex (DGC), which includes the sarcoglycans and sarcospan as well as several intracellular proteins (Lapidos et al., 2004). The DGC provides a mechanical link to the extracellular matrix, and mutations in genes involved in this multicomponent complex destabilize the muscle fiber plasma membrane and can lead to several different types of muscular dystrophy (Gao and McNally, 2015). *α*-dystroglycan is highly glycosylated (Ervasti and Campbell, 1991). This includes N- and O-linked modifications, in which oligosaccharides are attached to the amide group of asparagine, or to the hydroxyl group of serine or threonine respectively (Muntoni et al., 2007). O-mannosyl structures on dystroglycan have been proposed to be directly required for laminin, perlecan and agrin binding (Chiba et al., 1997; Sasaki et al., 1998; Michele and Campbell, 2003). Mutations in genes encoding for glycosyltransferases can cause certain forms of congenital muscular dystrophy characterized by abnormal glycosylation of *α*-dystroglycan (Muntoni et al., 2007). Increasing evidence suggests a direct role of dystroglycan in regulating MuSC function. It has been shown that muscle fiber specific deletion of *DAG1* leads to a mild form of muscular dystrophy (Cohn et al., 2002). Supporting the notion that *DAG1* is highly expressed in MuSCs, progenitors that fuse to knockout fibers induce its synchronized reexpression during regeneration cycles. Moreover, MuSC-specific deletion of *DAG1* has been shown to impair skeletal muscle regeneration (Dumont et al., 2015). Whether these effects are due to a reduced ability to maintain quiescence, or whether dystroglycan has a direct role following activation and proliferation, remains to be determined.

In skeletal muscle, heparan sulfate proteoglycans are present as integral matrix components such as perlecan, decorin, and biglycan. They can be attached to the ECM, as in the case of glypican, or can pass through the cell membrane, like syndecans (Iozzo et al., 1994; Brandan et al., 1996; Couchman and Woods, 1996; Wiberg et al., 2002). In MuSCs, syndecans-3 and -4 have important nonredundant roles in transducing and integrating signals from the ECM as well as from transmembrane signaling proteins (Cornelison et al., 2001). Loss of syndecan-3 leads to diminished proliferation, differentiation defects, and impaired self-renewal due to aberrant niche interactions, while loss of syndecan-4, which is highly expressed by MuSCs (Figures 2A,F), impairs activation, proliferation, migration, and differentiation *via* multiple mechanisms (Cornelison et al., 2004; Munoz et al., 2006; Pisconti et al., 2010; Shin et al., 2012; Bentzinger et al., 2013; Becsky et al., 2020; Ronning et al., 2020; Szabo et al., 2022). Through their extracellular domain and its associated heparan sulfate or chondroitin sulfate carbohydrate chains, syndecans form ternary structures with growth factors and their receptors including FGFs, transforming growth factor-β family members, Wnts, and small chemokines such as SDF-1/CXCL12 and RANTES/CCL5 (Allen et al., 2001; Slimani et al., 2003; Chen et al., 2004; Charnaux et al., 2005; Munoz et al., 2006). Syndecans also associate with transmembrane and membrane-bound ECM receptors such as integrins and A disintegrin and metalloproteases (ADAMs), and bind to extracellular matrix proteins including laminins, tenascin, collagens, and fibronectin (Couchman and Woods, 1999; Iba et al., 2000; Czarnowski, 2021). While some of these interactions are specific to only one syndecan, as for instance in case of syndecan-4, which is the sole and obligate coreceptor for CXCR4-CXCL12, many protein-protein interactions such as syndecan-integrin binding are common among all four family members, even though the four extracellular domains are each unique in their genomic sequence. Similarly, while with 28–34 amino acids their intracellular domains are comparably small, syndecans mediate interactions with a large number of second messengers, small G proteins, and cytoskeletal proteins (Simons and Horowitz, 2001). Syndecan-4 possesses a unique lysine-rich PIP2 binding domain that is not found in the other family members, which facilitates syndecan-4-specific signaling interactions. All members of the syndecan family form homodimers or multimers, which does not require their carbohydrate chains (Bernfield et al., 1992). In a process known as ectodomain shedding, the extracellular domains of syndecans can be proteolytically cleaved (Figure 2G) (Gopal, 2020). Syndecan shedding yields soluble proteoglycans retaining the binding characteristics of their transmembrane form that can become incorporated into the surrounding matrix and are even found in the systemic circulation (Eustace et al., 2019). Syndecan core protein cleavage by sheddases has been shown to be activated in response to cytokines such as IL-8 (Marshall et al., 2003). Multiple roles for syndecan shedding have been proposed, including a reduction of direct signal transduction in the originating cells, sequestration of growth factors away from cell surfaces, or broader paracrine effects on surrounding cells (Gopal, 2020). The plethora of ECM, cell-surface, and intracellular interactions that syndecans participate in highlights their roles as integrators of multiple signaling inputs and ‘tuners' of the downstream cellular response, although they are not generally considered to be either ECM components or classical transmembrane receptors. Recent single cell expression data further supports a role for syndecans, particularly syndecan-4, as key regulators of multiple signaling pathways in MuSCs (De Micheli et al., 2020). Expression of syndecan-4 is high in quiescent satellite cells, especially those that retain the most stem cell character and is maintained on proliferating and differentiating satellite cell progeny but lost on differentiated myofibers *in vivo* (Tanaka et al., 2009; Cho and Doles, 2017).

## Extracellular matrix in the quiescent muscle stem cell niche

**FIGURE 3:**
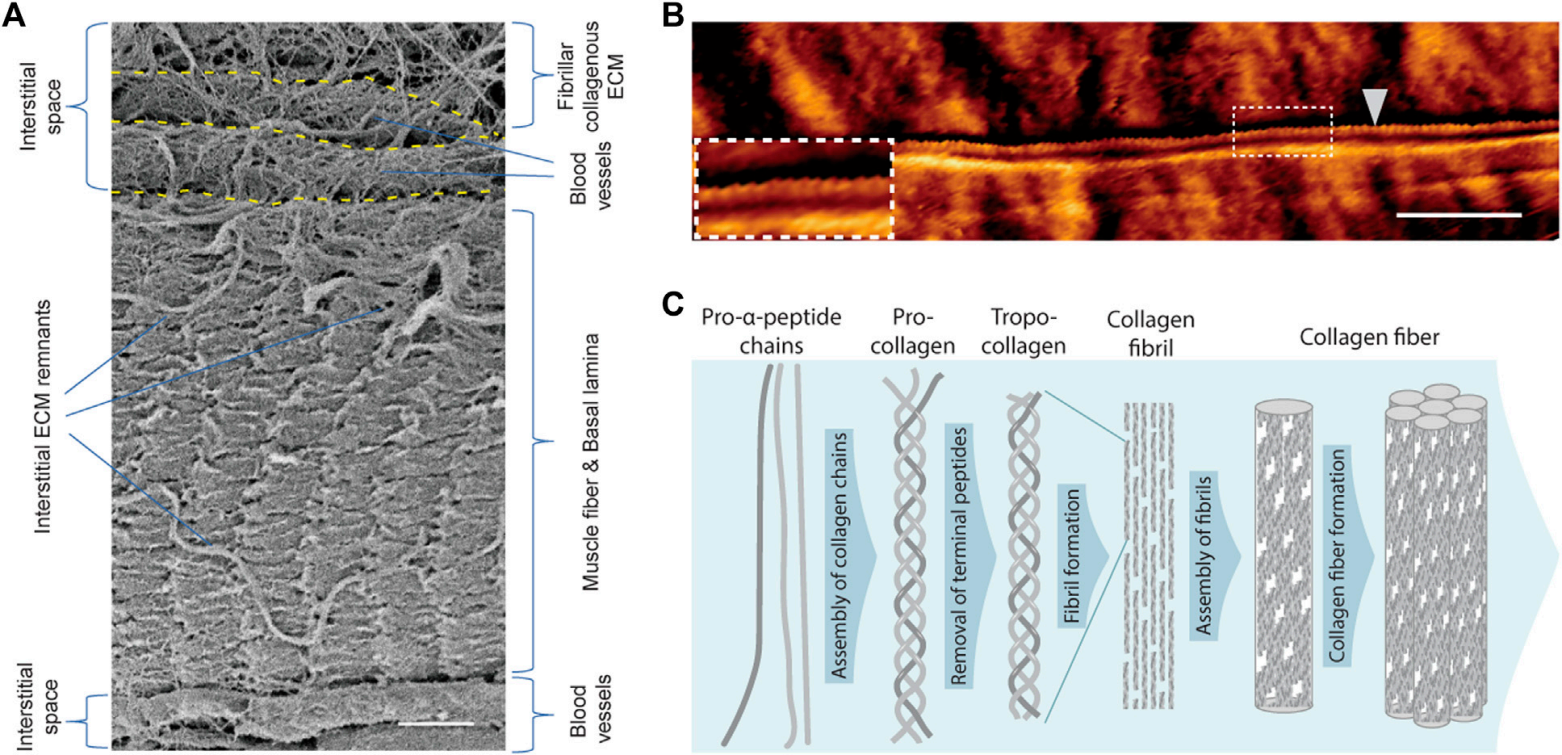
Fibrillar collagen and the interstitial space. **(A)** Scanning electron microscopy image of a manually teased mouse muscle fiber bundle preparation. The fiber is covered by the basal lamina and residues of interstitial ECM proteins. Flanking the fiber several ECM-covered blood vessels embedded in the interstitial space can be discerned. The upper most part of the image shows the extensive collagen I and III-rich ECM network in the interstitial space of skeletal muscle. **(B)** Atomic force microscopy image (contact mode) of a single collagen fibril (white arrowhead) on the surface of an enzymatically isolated single mouse muscle fiber. The characteristic banding pattern of overlap and gap regions of the collagen fibril can be discerned in the insert. **(C)** Scheme illustrating the fiber assembly process of collagen I. Scale bars = 2 µm **(A)**, and 1 µm **(B)**.

Collagen I and III fibrils are highly abundant in the interstitial space of homeostatic skeletal muscle (Figures 3A,B) (Light and Champion, 1984). Two genes, *COL1A1* and *COL1A2*, are involved in collagen I formation. Collagen I contains homo- or heterotrimers generated from the transcription of these genes, with each subunit comprising over 1000 amino acids that can reach a length of up to 300 nm (Naomi et al., 2021). Collagen III is a homotrimer encoded by the *COL3A1* gene that has the same molecular structure and approximate number of amino acids as collagen I (Kuivaniemi and Tromp, 2019). Collagen fibril formation is mainly an entropy-driven self-assembly process (Kadler et al., 1996). Fibril-forming collagens are synthesized in the form of soluble trimeric procollagen (Figure 3C). Through cleavage of N- and C-terminal peptide residues by metalloproteases, procollagen is processed into tropocollagen. Tropocollagen molecules spontaneously self-assemble into striated collagen fibrils that are stabilized by covalent bonds introduced by lysyl oxidase cross-linking.

In the endomysial space around muscle fibers collagen appears to be mainly organized in wavy fibrillar networks, while in the connective tissue of the perimysium surrounding bundles of muscle fibers, collagen can form large “cables” (Gillies and Lieber, 2011). These large collagen cables are oriented from tendon to tendon and run along the surface of muscle fibers. Interestingly, in a mouse model displaying extensive fibrosis, it has been shown that the number and not the size of collagen cables in the perimysium determines muscle stiffness (Gillies et al., 2017). Fibrillar collagens are major determinants of tissue elasticity, which has been demonstrated to be critical for the self-renewal capacity of MuSCs (Gilbert et al., 2010; Moyle et al., 2020; Piersma et al., 2020). MuSCs cultured on hydrogels with an elastic modulus around 12 kPa, corresponding to skeletal muscle tissue, show improved survival, reduced differentiation, and an increased engraftment capacity after transplantation (Gilbert et al., 2010). Notably, MuSC dysfunction in aged skeletal muscle correlates with a 4-fold increase in tissue stiffness, an accumulation of collagen, and a higher hydroxyproline and advanced glycation end-product content (Lacraz et al., 2015). Moreover, it has been shown that aged skeletal muscle is characterized by decreased collagen fibril tortuosity and alignment. (Stearns-Reider et al., 2017). The resulting increase in tissue stiffness leads to altered YAP/TAZ mechanosensing in fibroblasts. Consequently, these fibroblasts release an anti-myogenic ECM that increases the expression of fibrogenic markers in MuSCs.

**FIGURE 4:**
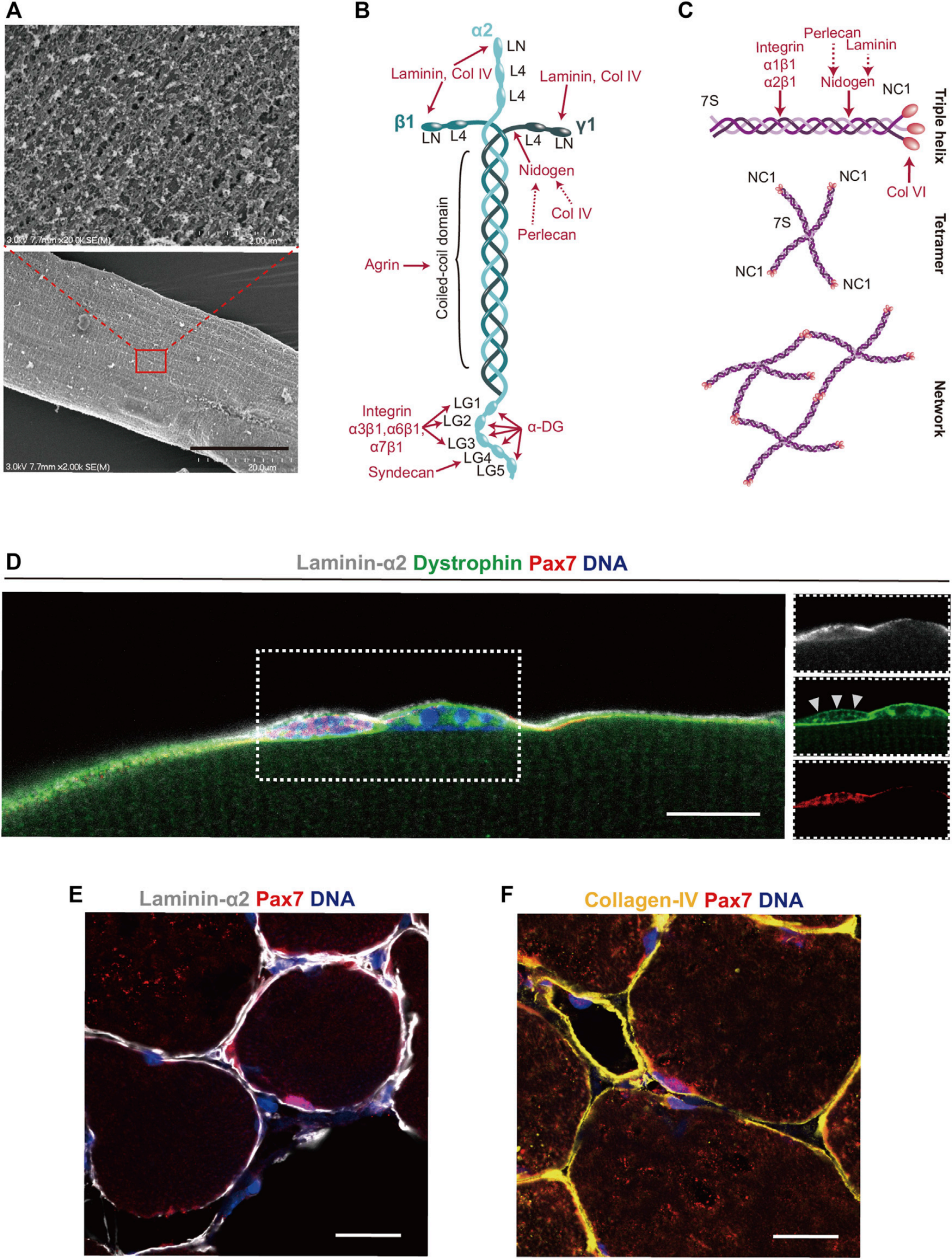
Laminin 211 and collagen IV in the basal lamina. **(A)** Scanning electron microscopy image of a single mouse muscle fiber that was isolated using collagenase type I, which cleaves collagen I, II, and III. The insert shows a higher magnification of the muscle fiber basal lamina as well as residues of interstitial ECM components. **(B,C)** Schemes showing the structure of laminin 211 and the assembly of the collagen IV network in the basal lamina. Interaction sites of ECM proteins and receptors are shown in red. **(D)** Immunostaining showing a single Pax7 positive MuSC (red) covered by laminin α2 (white) in the basal lamina, co-stained with the inner membrane marker dystrophin (green) and DNA (blue) on a manually teased mouse muscle fiber bundle. Dystrophin is expressed by both the muscle fiber and MuSCs, albeit levels are lower in quiescent MuSCs (white arrowheads). **(E)** Immunostaining of mouse skeletal muscle cross sections showing laminin α2 (white), **(E)**, collagen IV (yellow), **(F)**, Pax7 (red) and DNA (blue). Scale bars = 20 µm (Lower image A,D-F), and 2 µm (High magnification insert in A).

The popular single fiber isolation and culture method, which allows one to monitor MuSC dynamics *ex vivo*, exploits the high fibrillar collagen content in the interstitial space of skeletal muscle. In this technique, muscles are exposed to collagenase type I (ColG) typically isolated from the bacterium *Clostridium histolyticum*, which cleaves collagen I, II, and III (Rosenblatt et al., 1995; Cornelison and Wold, 1997; Pasut et al., 2013; Zhang et al., 2015). After digestion with this enzyme, single muscle fibers with their associated MuSCs and partially intact basal laminae that still contain most of their laminin and collagen IV core-networks can be isolated and maintained in growth media (Figure 4A). Due to its abundance of over 90% of the dry mass in bone and 60% in cartilage, collagen I from animal sources, either purified or in the form of gelatine, is frequently used as a coating substrate for the culture of MuSC derived primary myoblasts (Von Der Mark, 2006; Kim et al., 2020).

Notch signaling, which is for instance activated by proximity to blood vessels, controls enhancers proximal to the *COL5A1, COL5A3, COL6A1, and COL6A2* genes in quiescent MuSCs (Baghdadi et al., 2018a; Verma et al., 2018). MuSC-specific conditional deletion of *COL5A1* leads to premature cell cycle entry and a loss of quiescent cells from skeletal muscle. The quiescence promoting effects of collagen V appear to be mediated by the calcitonin receptor, a G-protein-coupled receptor that has been shown to have important functions in preventing MuSC activation *via* the cAMP-PKA pathway (Yamaguchi et al., 2015). Through microRNA-708, Notch signalling also has a role in supressing the expression of the focal adhesion protein tensin 3, which promotes MuSC quiescence and antagonizes cell migration (Baghdadi et al., 2018b). Tensins integrate ECM signals by binding to the intracellular tail of integrin β1, as well as to the actin cytoskeleton (Mouneimne and Brugge, 2007). Interestingly, Pax3-dependent deletion of the Notch effector Rbpj has been shown to lead to decreased autocrine deposition of laminin and collagen XVIII around emerging MuSCs during development (Brohl et al., 2012). Thus, several lines of evidence point towards Notch signaling as a key upstream regulator of cell-autonomous ECM deposition in the MuSC niche.

Over 30 years ago laminin was identified and isolated from Engelbreth-Holm-Swarm (EHS) mouse sarcoma (Chung et al., 1979; Timpl et al., 1979). Laminins are heterotrimeric high molecular weight proteins (∼400–900 kDa) that contain *α*, *β,* and *γ* chains, which depending on the subunit isoforms assemble into cross- or T-like structures (Holmberg and Durbeej, 2013). The five *α*, four *β*, and three *γ* chains in mammals are believed to have evolved from a single archetypical set of genes (Domogatskaya et al., 2012). Even though the laminin genes allow for over 60 possible permutations, only 16 heterotrimer combinations have been identified (Yap et al., 2019). Laminins are named based on the chains they contain. For instance, laminin 111, the isoform most abundantly secreted by EHS sarcoma cells, contains α1β1γ1 chains (Aumailley et al., 2005). αβγ laminin chains assemble through their coiled coil domains that are linked to each other by disulphide bonds (Hohenester, 2019).

During development the skeletal muscle basal lamina contains high amounts of laminin 211, while the 411 and 511 isoforms are detectable at much lower levels (Sanes et al., 1990; Patton et al., 1999). Except for the neuromuscular junction and blood vessels, the basal lamina in homeostatic adult skeletal muscle contains almost exclusively laminin 211. Following regeneration laminin 511 is transiently upregulated, while the 211 and 411 isoforms remain largely unchanged (Patton et al., 1999). The autopolymerisation of laminin 211 depends on the N-terminal globular LN domains of the three short arms (Figure 4B). Isoforms of laminin containing the α4, α3A, and γ2 chains lack the N-terminal globular domains and do not polymerize efficiently (Karamanos et al., 2021). Laminins mostly bind cell-surface receptors through five G domains (LG1–5) on the *α* chain C-terminus. While LG1–3 are responsible for binding to integrin α7β1, binding to dystroglycan appears to involve all 5 LG domains (Talts et al., 1999; Timpl et al., 2000; Smirnov et al., 2002). Due to its abundance in the niche, laminin 211 likely has a role in maintaining MuSC quiescence (Figures 4D,E). Supporting this notion, it has been shown that primary MuSC derived myoblasts proliferate and differentiate less efficiently on laminin 211 than on laminins containing the α5 chain (Penton et al., 2016).

Several peptide motifs in the LG4 domain of the laminin α2 chain contain heparin-binding sites and interact with syndecans, which could be another mechanism involved in the maintenance of MuSC quiescence (Hoffman et al., 1998; Suzuki et al., 2003). The N-terminal domain (NtA) of agrin binds to laminin near the center of its coiled coil domain and, through some of its C-terminal laminin-like G domains, links the basal lamina to cell surface receptors such as dystroglycan (Gesemann et al., 1996; Denzer et al., 1998). Nidogen-1 and -2 (also known as entactins) are 30–40 nm long sulfated monomeric glycoproteins of about 150 kDa that have a structurally homologous domain organisation (Kohfeldt et al., 1998). Nidogen has two N-terminal and one C-terminal globular domains that are connected by rod-like segments (Fox et al., 1991). By providing a link between the laminin and collagen IV networks, nidogen represents an important central hub in the basal lamina ECM (Mayer et al., 1993; Reinhardt et al., 1993; Kohfeldt et al., 1998). Binding of nidogen to the triple helical rod domain of collagen IV occurs *via* its G2 domain (Aumailley et al., 1989; Aumailley et al., 1993). Next to the collagen IV network, nidogen also connects the large proteoglycan perlecan to laminin (Hopf et al., 1999). The perlecan core protein has a molecular weight of approximately 500 kDa, which is increased by the addition of multiple heparan sulfate chains (Farach-Carson and Carson, 2007). Perlecan knockout mice show changes in the number of fast contracting fibers and display muscle hypertrophy accompanied by decreased myostatin expression, but direct effects on MuSCs remain to be assessed (Xu et al., 2010). Notably, perlecan binds and enhances the activity of several growth factors including vascular endothelial growth factor (VEGF) and fibroblast growth factors (FGFs) (Jiang and Couchman, 2003). Since VEGF is involved in maintaining quiescence and FGFs inhibit differentiation, it is plausible that perlecan mediated tethering of these molecules in the basal lamina plays a role in regulating MuSC function (Pawlikowski et al., 2017; Verma et al., 2018).

Collagen IV, which next to laminin forms the second core network of the basal lamina, originates from up to six different *α*-chains named α1(IV) to α6(IV) that can assemble into α1α1α2, α3α4α5, and α5α5α6 heterotrimers (Khoshnoodi et al., 2008). The α1(IV) and α2(IV) chains are present in most adult tissues, while the other four chains appear to largely play a role during development. Collagen IV chains contain an N-terminal 7S domain (named based on its sedimentation coefficient) that is followed by a long collagenous domain, and a non-collagenous C-terminal domain (NC1) (Figures 4C,F) (Hudson et al., 2003). By binding of two NC1 domains or by uniting four triple helical 7S domains, collagen IV can form irregular polygonal networks (Yurchenco and Ruben, 1987). Mutations in *COL4A1* have been shown to correlate with myopathic changes in skeletal muscles of a human patient and mice with a mutation in this gene develop a progressive neuromuscular phenotype (Labelle-Dumais et al., 2019). However, a direct role of collagen IV in regulating MuSC function remains to be uncovered. *In vitro* experiments using blocking antibodies suggest that MuSC binding to collagen IV is largely indirect and mediated by perlecan (Villar et al., 1999). Cross-linking of NC1 domains in collagen IV is established through sulfilimine bonds (between methionine sulfur and lysine nitrogen), whose loss has been shown to influence tissue elasticity in the kidney (Bhave et al., 2017). Thus, it is conceivable that alongside interstitial fibrillar collagens, the collagen IV network influences MuSC function by finetuning the biomechanical properties of the basal lamina and the tissue in general (Gilbert et al., 2010).

## Extracellular matrix regulation of activated muscle stem cells

Following damage to muscle fibers, MuSCs exit quiescence and enter the cell cycle to subsequently initiate the regenerative response that involves a multitude of highly complex spatiotemporally controlled niche interactions with a wide range of different supportive cell types (Mashinchian et al., 2018). In earliest stages of MuSC activation cellular protrusions stabilized by Rac GTPases are retracted through an upregulation of Rho/ROCK signaling, which is correlates with an increase in the expression of immediate early gene products such as c-jun and c-fos (Machado et al., 2017; van Velthoven et al., 2017; Almada et al., 2021; Kann et al., 2022). In order to overcome inhibitory signals by pro-inflammatory immune cells following skeletal muscle injury, the histone H3 lysine 27 demethylase JMJD3 epigenetically modifies the Has2 locus in MuSCs, which initiates hyaluronic acid synthesis and creates a permissive autoregulatory niche allowing the cells to exit quiescence (Nakka et al., 2022). Intravital imaging of mouse skeletal muscle has revealed that MuSC division and migration following injury are mostly oriented bi-directionally along the longitudinal axis of remnant basal lamina sheets that were termed “ghost fibers” (Webster et al., 2016). Reorientation of these ghost fibers lead to disorganization of newly formed muscle fibers, which emphasizes the importance of an instructive ECM template during the early stages of the regenerative response.

**FIGURE 5:**
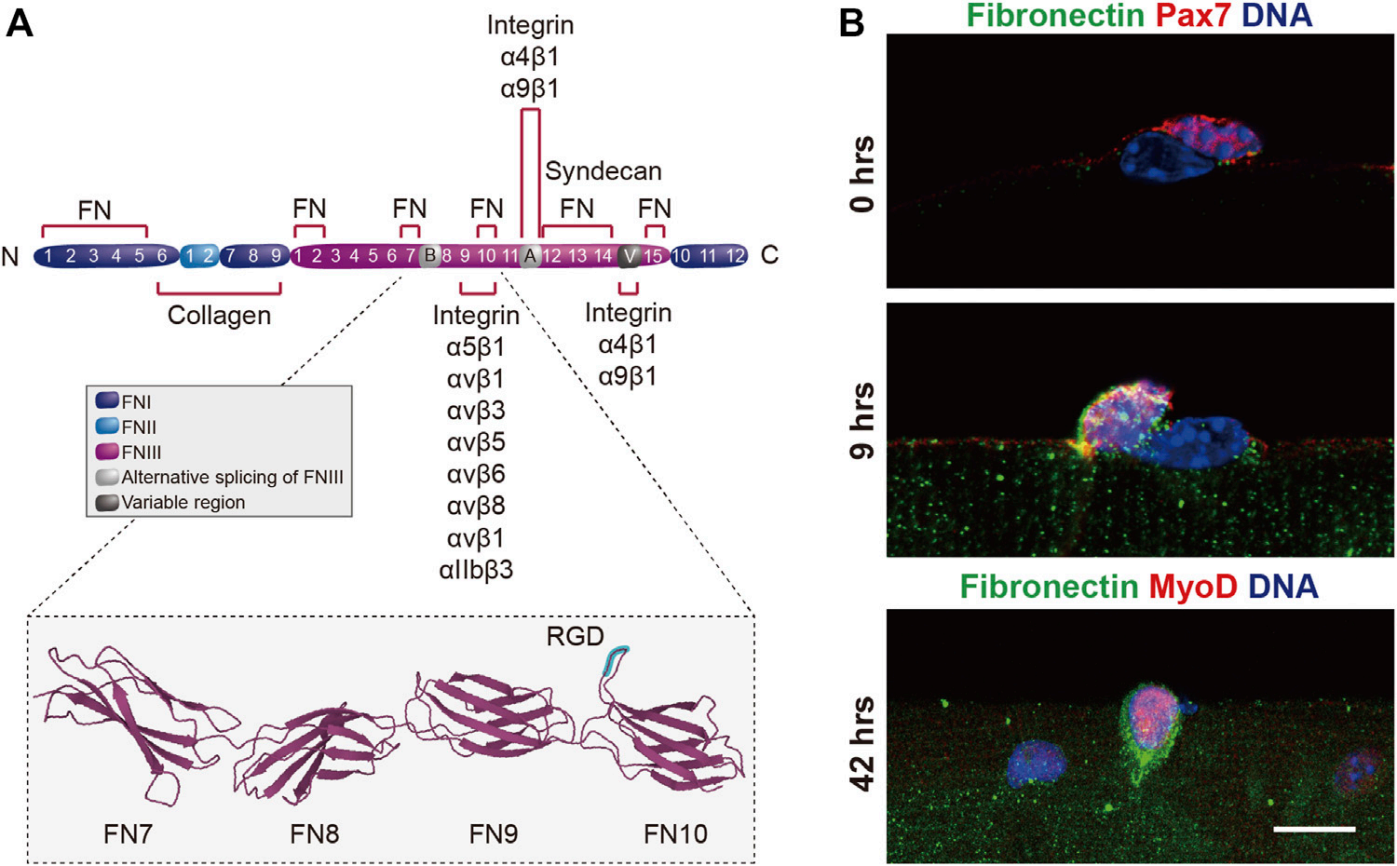
Autologous expression of fibronectin by MuSCs. **(A)** Scheme showing the domain structure of fibronectin and the binding sites for collagen, integrin and syndecan. The insert shows the seventh through the RGD-containing 10th type III repeats of fibronectin (Leahy et al., 1996) obtained from the RCSB Protein Data Bank (Berman et al., 2000). The RGD motif is highlighted in blue. **(B)** Immunostaining showing the endogenous expression of fibronectin (green), Pax7 or MyoD (red) and DNA (blue) in muscle stem cells on enzymatically isolated mouse single muscle fibers after 0, 9, and 42 h (hrs) in culture. Scale bar = 20 µm.

Within 8–9 h after activation, MuSCs begin to secrete copious amounts of the ECM glycoprotein fibronectin into their microenvironment (Figures 5A,B) (Bentzinger et al., 2013). Fibronectin has a molecular weight of about 230–270 kDa and is typically found as a dimer connected by C-terminal disulfide bonds (Singh et al., 2010). The fibronectin molecule consists of 12 FNI, 2 FNII, and 15–17 FNIII domains and, depending on alternative splicing, three main variable domains, the EDA and EDB FNIII extra domains, and the IIICS region can be included or excluded. Fibronectin is either found as soluble plasma fibronectin (pFN) devoid of EDA and EDB domains, or as cellular fibronectin (cFN) that may contain one or both of the EDA and EDB extra domains. Next to its ability to bind to laminin and collagen, fibronectin can engage a variety of integrins as well as syndecan-4 (Mao and Schwarzbauer, 2005). The RGD sequence (Arg–Gly–Asp) of fibronectin, which binds to α5β1 and αVβ3 integrins, is found in the 10th FNIII domain. Fibronectin is required for normal skeletal muscle regeneration and genetic ablation in mice leads to reduced MuSC numbers (Bentzinger et al., 2013; Lukjanenko et al., 2016). Whether specific splice variants of fibronectin have differential roles during skeletal muscle regeneration has not yet been explored. The expression of connective tissue growth factor (CTGF/CCN2), which is implicated in modulating TGF-β activity, has been shown to be an autoregulatory upstream regulator of fibronectin expression in activated myogenic cells (Vial et al., 2008). CTGF signalling is deregulated in pathologic conditions that are accompanied by excessive fibrotic deposition of ECM. Next to cell-autonomous secretion by activated MuSCs, fibronectin is secreted by fibro–adipogenic progenitors (FAPs) and cells of the hematopoietic lineage in regenerating skeletal muscle (Lukjanenko et al., 2016). Fibronectin binds to the syndecan-4/frizzled7 co-receptor complex and, together with Wnt7a, expands the stem cell pool during muscle regeneration (Bentzinger et al., 2013). As a consequence of aging, fibronectin levels are significantly reduced in regenerating skeletal muscle and exogenous supply of this protein improves MuSC function (Lukjanenko et al., 2016). Reduced levels of fibronectin in aged muscles goes along with a decreased activation of integrin β1 in MuSCs, which has been discussed in more detail above (Rozo et al., 2016).

Laminin α1 and α5 have been shown to be enriched in the microenvironment of activated MuSCs (Rayagiri et al., 2018). Sox2-dependent ablation of laminin α1 in mice leads to a reduction in sublaminal MuSCs and a shift towards smaller muscle fibers in a multiple injury paradigm. MuSCs also express collagen VI and have been shown to reside in a microenvironment enriched in this ECM component (Urciuolo et al., 2013). Collagen VI is implicated in the pathogenesis of some forms of muscular dystrophy and is required for MuSC self-renewal, maintenance and survival. During development fetal myogenic progenitors have been shown to express elevated levels of tenascin-C, fibronectin, and collagen VI. In transplantation experiments it was observed that all three of these ECM components are critical for the function of adult MuSCs, while only knockdown of tenascin-c and collagen VI reduced the ability of fetal myogenic progenitors to participate in skeletal muscle repair (Tierney et al., 2016). Next to autoregulatory ECM deposition, activated MuSCs also remodel their structural microenvironment enzymatically. This includes expression of matrix metalloproteinase (MMP) 2 and 9 (Rayagiri et al., 2018). Pharmacologic inhibition of MMPs impairs MuSC proliferation in single fiber culture and genetic ablation of MMP9, which is pathologically increased in a mouse model of Duchenne muscular dystrophy, improves muscle regeneration (Li et al., 2009). Human myogenic progenitors express MMP14, which has been shown to be required for invasion of collagen I matrices (Lund et al., 2014). In summary, these results support the idea that autocrine deposition and modulation of different ECM components has stage-specific regulatory roles in the developmental and adult MuSC niche.

**FIGURE 6:**
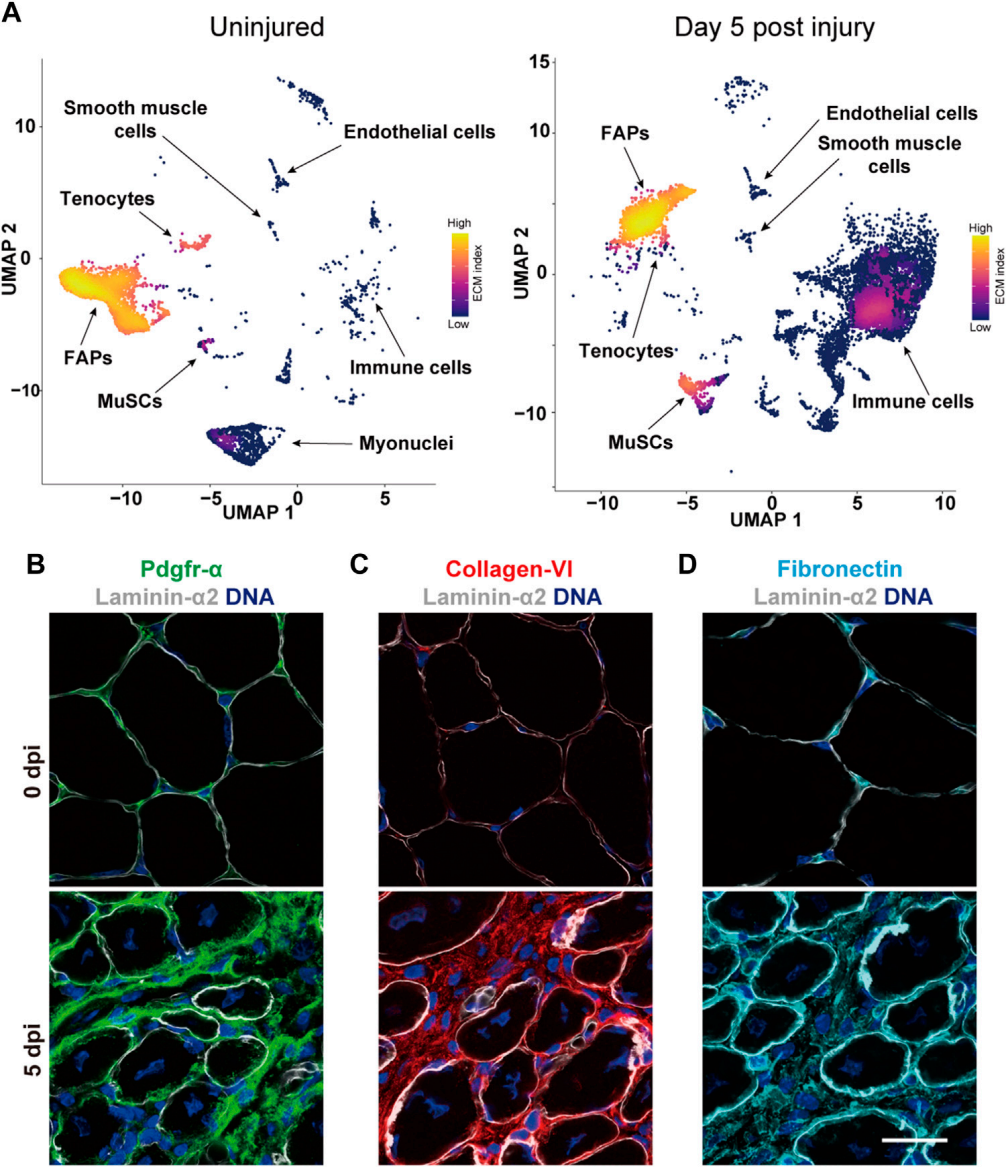
The MuSC niche in regenerating skeletal muscle. **(A)** The images show the joint gene expression density (Alquicira-Hernandez and Powell, 2021) of an index of ∼100 ECM genes obtained from the Matrisome database (Shao et al., 2020) in the UMAP projection (McInnes et al., 2018) of single cell RNA-sequencing data (Oprescu et al., 2020) deposited in the Gene Expression Omnibus (GEO) database (Barrett et al., 2013) from uninjured and regenerating mouse skeletal muscle at 5 days post injury. FAPs is short for fibro–adipogenic progenitors. **(B)** Immunostaining of mouse skeletal muscle cross sections under uninjured conditions (0 dpi) or 5 days post injury (5 dpi) visualizing FAPs based on Pdgfrα (green) co-stained with laminin α2 (white) and DNA (blue). **(C,D)** Immunostaining of mouse skeletal muscle cross sections at 0 or 5 dpi showing collagen IV (red), fibronectin (turquoise), laminin α2 (white) and DNA (blue). Scale bar = 20 µm.

Amongst all mononuclear cell types contributing to skeletal muscle regeneration, FAPs appear to secrete the most significant amounts of ECM into the tissue (Figures 6A,B) (De Micheli et al., 2020; Oprescu et al., 2020). Large-scale integration of single-cell transcriptomic data sets has revealed that FAPs have a predicted interaction strength with myogenic cells that is magnitudes higher than with any other mononuclear cell type in regenerating muscle (McKellar et al., 2021). Moreover, 58% of the interactions between FAPs and MuSCs fall into the “Secreted Signaling” category, while 36% involve “ECM-Receptor” interactions. In agreement with their classification as mesenchymal stromal cells and coinciding with their anatomic localization, FAPs in homeostatic adult skeletal muscle express high levels of the interstitial ECM components collagen III and decorin (De Micheli et al., 2020). Close to the peak of MuSC proliferation around day 5 post injury, FAPs show strong expression of collagen I alongside periostin, biglycan and SPARC. Moreover, it has been demonstrated that a population of connective tissue resident mesenchymal cells are the main cell type secreting collagen VI in skeletal muscle (Figure 6C) (Braghetta et al., 2008). During development myogenic cells are required to activate a *COL6A1* gene enhancer region in connective tissue resident mesenchymal cells. Notably, it has been shown that diphtheria-toxin mediated ablation of MuSCs in adult skeletal muscles leads to an expansion of mesenchymal fibroblast-like cell types and substantial fibrosis (Murphy et al., 2011). These observations suggest that MuSCs and ECM secreting mesenchymal cell populations have an intimate reciprocal relationship during skeletal muscle regeneration. FAPs have also been shown to be affected by the aging process and produce lower levels of the ECM-associated matricellular signaling protein WISP1, which influences MuSC self-renewal and differentiation. Transplantation of young FAPs or treatment with WISP1 restores the myogenic capacity of MuSCs in aged mice and improves skeletal muscle regeneration (Lukjanenko et al., 2019). Aged FAPs also secrete higher levels of SPARC Related modular calcium binding 2 (Smoc2), another matricellular protein, which induces aberrant integrin and MAP kinase signaling in aged MuSCs (Schüler et al., 2021).

During the expansion phase of the MuSC pool following injury, skeletal muscle becomes permeated with a transitional pro-regenerative ECM environment that is fundamentally different from its homeostatic composition (Goetsch et al., 2003; Bentzinger et al., 2013; Ceafalan et al., 2020). By day 5 after snake venom injury around 50% of transcripts that are at least two-fold increased in regenerating skeletal muscle fall into the gene ontology category of “ECM signaling” (Goetsch et al., 2003). This pro-regenerative ECM involves high levels of biglycan, fibronectin, collagen I and III-VIII, tenascin C, agrin, laminin α2, α5, β1, and β2, nidogen, perlecan, periostin, and others. Interestingly, during the remarkably efficient limb regeneration process in urodele amphibians a transitional regenerative ECM containing hyaluronic acid, fibronectin, and tenascin C has been suggested to instruct cell cycle entry and proliferation of myogenic progenitors (Calve et al., 2010). Under homeostatic conditions, these ECM molecules are thought to localize mainly in the interstitial space (Frantz et al., 2010). However, during the appendage regeneration process some of them can be detected in the basal lamina of muscle fibers, which suggests that they have a direct role in regulating the function of sublaminal myogenic progenitors. In regenerating mouse skeletal muscles, a similar phenomenon can be observed. While certain typically interstitial ECM components such as collagen VI do appear to overlap to a significant degree with the basal lamina during skeletal muscle regeneration, others such as fibronectin permeate it extensively (Figures 6C,D). Together with the observation that activated sublaminal MuSCs express several typically interstitial molecules with autoregulatory function, these observations suggest that the traditional compartmentalization of ECM components applies only partially during skeletal muscle regeneration.

Several ECM components have been implicated in the regulation of MuSC differentiation. For instance, the small proteoglycan fibromodulin antagonizes the inhibitory effects of myostatin and promotes myoblast differentiation (Lee et al., 2016). Insulin-like growth factor 1 stimulates myoblast fusion in synergy with collagen IV (Ito et al., 2015). The large glycoprotein fibrillin-1 is yet another example of an ECM component that influences myogenesis by modulating growth factor activity. Mutations in fibrillin-1 cause the connective tissue disease Marfan syndrome and influence the bioavailability of TGFβ1 (Chaudhry et al., 2007). Mice with a mutation in fibrilin-1 analogous to that found in humans with Marfan syndrome, display chronically increased TGFβ1 signaling, which impairs muscle regeneration by inhibiting MuSC proliferation and differentiation (Cohn et al., 2007). The heparan sulfate proteoglycan glypican-1 is expressed by MuSC-derived myoblasts and sequesters FGF-2 into lipid rafts, allowing the cells to efficiently differentiate. Loss of glypican-1 leads to reduced myotube formation and supresses the expression of myogenin (Gutierrez and Brandan, 2010). An analogous pro-differentiative mechanism seems to involve perlecan, which is also expressed by myoblasts and binds FGF-2 (Larrain et al., 1997). Activated MuSCs also express reversion-inducing cysteine-rich protein with Kazal motifs (RECK), an extracellular GPI-anchored glycoprotein with inhibitory functions for matrix metalloproteinases (Gutierrez et al., 2021). Reduced levels of RECK increase differentiation both *in vitro* and in knockout mice. These observations suggest a role of this protein in fine-tuning of the differentiation process by ECM remodeling. Altogether, multiple ECM proteins are involved in controlling MuSC differentiation and, in particular, the regulation of growth factors and matrix remodelling enzymes appears to be of central importance during this process.

## Extracellular matrix-related muscular dystrophies and muscle stem cells

Muscular dystrophies are genetic disorders characterized by skeletal muscle weakening and degeneration that can lead to severe disability and premature death. Increasing evidence suggests that MuSC dysfunction plays a role in driving the progressive regenerative failure that characterizes many of these diseases (Le Moal et al., 2022). Recently, the term “satellite cell-opathies” has been proposed to describe conditions in which MuSC defects are the principal driver of disease progression (Ganassi et al., 2022). For instance, mutations in the MuSC master regulator Pax7 have been shown to lead to a progressive congenital myopathy in humans (Feichtinger et al., 2019; Marg et al., 2019). In case of muscular dystrophies caused by mutations that lead to an instability of muscle fibers, regenerative failure and MuSC dysfunction can be concomitant or arise subsequent to disease-initiating events. Consequently, such conditions may be considered secondary satellite cell-opathies. An example is Duchenne muscular dystrophy, a condition characterized by mutations in the DGC protein dystrophin, an inner membrane protein that is expressed by both muscle fibers and MuSCs. In preclinical models of Duchenne muscular dystrophy, it has been shown that in addition to the typical chronic multifocal muscle fiber degeneration, asymmetric satellite cell division is impaired and leads to a decreased pool of committed progenitors (Dumont et al., 2015).

Several other mutations causing forms of muscular dystrophy affect gene products involved in linking the interstitial space and the basal lamina to the muscle fiber plasma membrane (Henry and Campbell, 1999; Guiraud et al., 2015; Gawlik, 2018). In particular, mutations in the *LAMA2* gene coding for the laminin α2 chain cause LAMA2-related muscular dystrophy, while mutations in the *COL6A1, COL6A2, and COL6A3* genes lead to collagen VI-related myopathies, including Bethlem myopathy and Ullrich congenital muscular dystrophy (Pegoraro et al., 2000; Allamand and Guicheney, 2002; Lampe and Bushby, 2005; Bonnemann, 2011). The critical role of both laminin α2 and collagen VI in regulating MuSCs, supports the notion that stem cell dysfunction contributes to disease pathogenesis in both types of muscular dystrophy (Le Moal et al., 2022).

**FIGURE 7:**
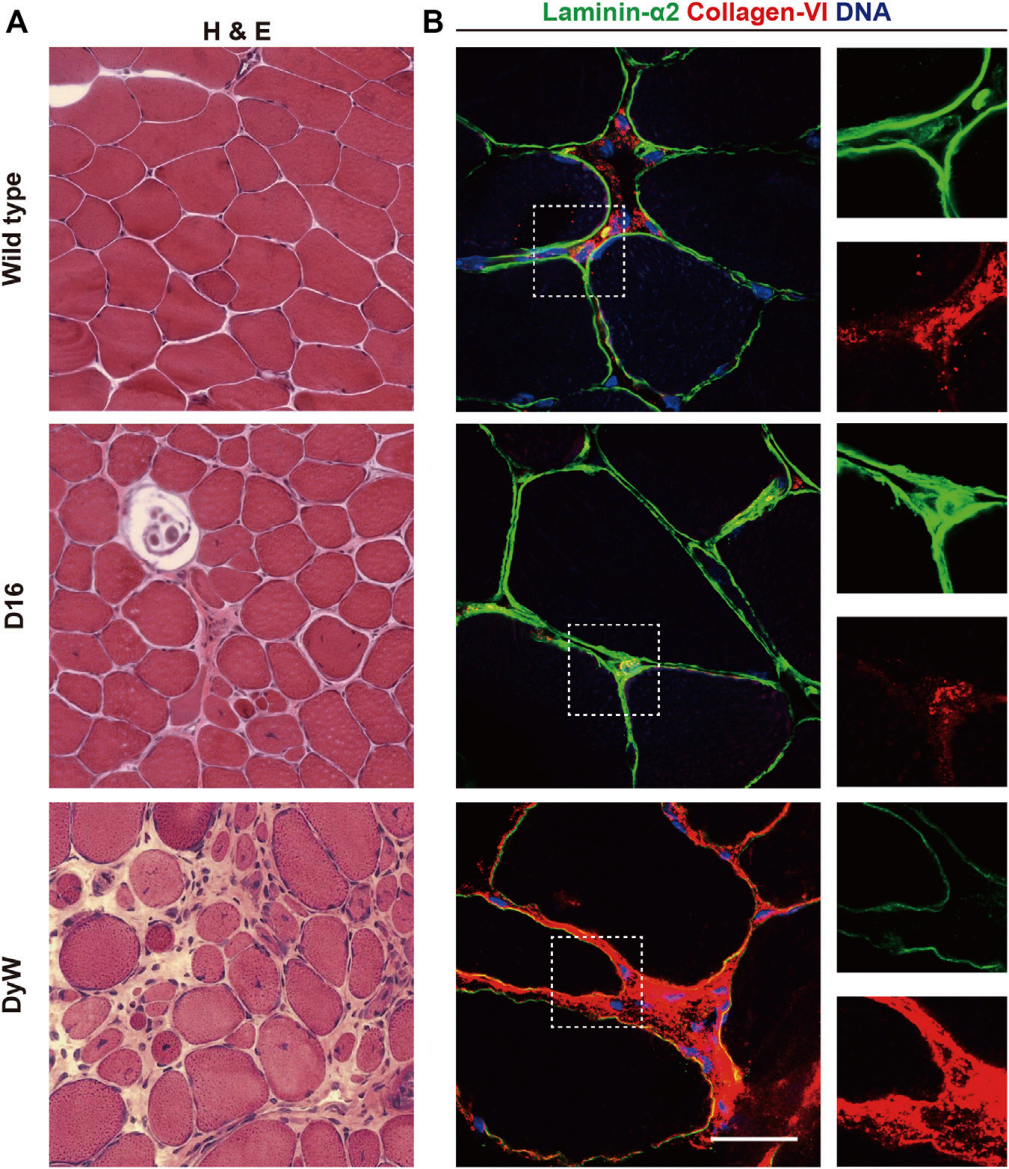
ECM and muscular dystrophy. **(A)** Hematoxylin and eosin (H&E) staining of skeletal muscle sections from wild type mice, *COL6A3* mutant D16 mice, and *LAMA2* mutant dyW mice (Kuang et al., 1998; Pan et al., 2014). **(B)** Immunostaining of skeletal muscle cross sections from wild type, D16 and dyW mice showing laminin α2 (green), collagen VI (red) and DNA (blue).

LAMA2-related muscular dystrophy shows a spectrum of clinical manifestations ranging from comparably mild late onset forms in adulthood to conditions that manifest in neonates, leading to severe muscle wasting accompanied by a broad spectrum of secondary effects such as spinal deformities (Sarkozy et al., 2020). Representative of the spectrum of disease severity in humans, several mouse models of LAMA2-related muscular dystrophy have been generated that express varying levels of mutated *LAMA2* gene products or are full knockouts (Gawlik and Durbeej, 2020). These animal models reproduce the human skeletal muscle pathology well and present with features such as a wide variation in fiber size, extensive regeneration evidenced by centralized myonuclei, and fibrosis (Figures 7A,B). Interestingly, in the dyW model, which expresses reduced levels of a truncated LAMA2 gene product, or in dy3K mice which are complete knockouts, levels of the laminin receptor *α*-dystroglycan are significantly reduced (Moll et al., 2001; Bentzinger et al., 2005). In agreement with an important role of the laminin-dystroglycan interaction in MuSCs, dyW, and dy3K mice show incomplete skeletal muscle regeneration (Kuang et al., 1999; Bentzinger et al., 2005; Le Moal et al., 2022). Loss of the laminin α2 chain in LAMA2-related muscular dystrophy patients and mutant mice leads to a compensatory upregulation of laminin α4, which binds only weakly to dystroglycan and integrin α7β1, and does not effectively autopolymerize (Patton et al., 1997; Ringelmann et al., 1999; Colognato and Yurchenco, 2000; Talts et al., 2000; von der Mark et al., 2002; Reinhard et al., 2017). During fetal development in dyW mice laminins 411 and 511 are both present in close proximity to myogenic progenitors (Nunes et al., 2017). However, these laminins do not seem to be able to compensate for laminin α2, and the mice display reduced fetal muscle growth accompanied by a lower number of Pax7 and myogenin positive cells. Notably, restoration of basal lamina polymerization and cell surface receptor binding by transgenic expression chimeric linker-molecules composed of domains of agrin, nidogen, and laminin α1 leads to a dramatic rescue of the dystrophic pathology in dyW mice (Moll et al., 2001; Reinhard et al., 2017). One of these transgenes, a miniaturized form of agrin containing binding sites for laminins and *α*-dystroglycan, significantly improves skeletal muscle regeneration in dy3K mice (Bentzinger et al., 2005). Interestingly, it has been shown that laminin 111 is able to reduce pathology in mouse models of Duchenne and LAMA2-related muscular dystrophy (Rooney et al., 2009; Rooney et al., 2012). Presumably laminin 111 stabilizes dystrophin deficient muscle fibers *via* integrin α7, while it compensates for multiple processes in laminin α2 deficient muscles. In light of its important function for MuSCs, Laminin 111 likely also improves the regenerative capacity of *LAMA2* deficient muscles (Rayagiri et al., 2018).

Similar to LAMA2-related diseases, collagen VI-related myopathies show a continuum of phenotypes ranging from comparably mild Bethlem myopathy to severe Ullrich congenital muscular dystrophy (Allamand et al., 2011; Bonnemann, 2011). A spectrum of different mutations in the *COL6A1, COL6A2* and *COL6A3* genes have been shown to cause effects ranging from dominant negative mechanisms to complete loss of function (Allamand et al., 2011; Bonnemann, 2011). The most common clinical presentation of collagen VI-related myopathies is muscle weakness, contractures, and hyperlaxity (Allamand et al., 2011). In Ullrich congenital muscular dystrophy these symptoms can be very severe and lead to an arrest of motor milestones. Skeletal muscle in Ullrich muscular dystrophy patients displays dystrophic features including fiber size variation, fibrosis, and centralized myonuclei (Nonaka et al., 1981). Mouse models of collagen VI-related myopathies show comparably mild phenotypes, which have paradoxically made them highly useful in exploring the underlying disease mechanisms (Figure 7A) (Bonaldo et al., 1998; Pan et al., 2014). In both *COL6A1* and *COL6A3* mutant mice, mild myopathic changes are associated with ultrastructural changes in mitochondria and the sarcoplasmic reticulum (Irwin et al., 2003; Pan et al., 2014). In addition, defects in autophagic flux in *COL6A1*-deficient mice have been linked to apoptotic processes in skeletal muscle (Grumati et al., 2010). *COL6A1*-deficient mice show smaller fibers and lower numbers of MuSCs after snake venom injury, and have a reduced ability to maintain quiescent MyoD negative cells (Urciuolo et al., 2013). In *COL6A1* deficient mice, wild-type MuSCs have been shown to engraft better than *COL6A1*-deficient cells, suggesting that this ECM component acts through a cell-autonomous mechanism. Interestingly, the authors also demonstrated that *COL6A1*-deficient muscles display an increase in tissue elasticity whose partial rescue by transplantation of wild-type fibroblasts ameliorates the MuSC defect. Similarly, MuSC pathology in *COL6A1* deficient mice is reduced upon transplantation of collagen VI secreting human adipose-derived stem cells and mesenchymal stromal cells (Alexeev et al., 2014; Takenaka-Ninagawa et al., 2021). Thus, collagen VI-related myopathies are another example highlighting the importance of tissue elasticity in the pathogenesis of MuSC defects.

## Concluding remarks

Recent advances in single cell RNA sequencing have considerably improved our understanding of the contribution of different cell types to ECM synthesis in skeletal muscle and the MuSC niche. One interesting problem associated with single cell sequencing is the fact that muscle fibers are multinucleated, and therefore difficult to analyze using this technique. Thus, except for insights obtained from single nucleus RNA sequencing experiments, which only cover a fraction of all transcripts, the proportional contribution of muscle fibers to ECM synthesis in the niche remains somewhat enigmatic. In particular, newly formed multinucleated muscle fibers are highly transcriptionally active and may well contribute significantly to the pro-regenerative transitional ECM that instructs MuSC function following skeletal muscle injury. Emerging technologies such as spatial RNA-sequencing, multiplexed proteomics, and single-cell protein analysis, will ideally further advance our understanding of the complex niche regulation of MuSCs (Pham et al., 2021; McKellar et al., 2022; Wang et al., 2022). Importantly, compared to other relatively static stem cell niches, the microenvironment of MuSCs is highly dynamic and depends on the respective stage of muscle regeneration. Many aspects of the intricate ECM biology involved in these processes, for instance the return of MuSCs to quiescence, remain underexplored and future research regarding these topics will undoubtedly open interesting new avenues for stem cell dysfunction in aging and disease.
